# Celiac Disease

**DOI:** 10.1155/2012/758560

**Published:** 2012-08-16

**Authors:** Govind K. Makharia, Carlo Catassi, Kheen Lee Goh, C. J. J. Mulder

**Affiliations:** ^1^Department of Gastroenterology and Human Nutrition, All India Institute of Medical Sciences, New Delhi, India; ^2^Clinica Pediatrica, Università Politecnica delle Marche, Ancona, Italy; ^3^Center for Celiac Research, University of Maryland School of Medicine, Baltimore, USA; ^4^Department of Gastroenterology, University of Malaya Medical Center, University of Malaya, Kuala Lumpur, Malaysia; ^5^Department of Gastroenterology, VU University Medical Center, Amsterdam, The Netherlands

Celiac disease is a chronic systemic autoimmune disorder induced by gluten proteins present in wheat, barley, and rye. Celiac disease was originally described in 19th century principally in children by Samuel Gee in England and by Christian Herter in the United States. Until the mid-20th century, celiac disease was known as Gee-Herter disease. About two decade ago, celiac disease was considered rare outside Europe and, therefore, was almost completely ignored by health care professionals in rest of the world. The initial diagnostic criteria laid down by the European Society of Pediatric Gastroenterology and Nutrition in 1970 required three sets of intestinal histological evaluation, that is, demonstration of villous atrophy on gluten containing diet, normalization of villous atrophy on gluten-free diet (GFD), and reappearance of villous atrophy on gluten re-challenge. After 1990, relaxation of the diagnostic criteria (reduction in number of biopsies required for the diagnosis from three to one), increase in awareness, and availability of the serological tests; celiac disease in the past 15–20 years has become a global disease and has moved from obscurity into the popular spotlight worldwide. 

It was initially thought that gluten hypersensitivity in celiac disease is limited to intestine only and all other features are secondary to malabsorption,but it is now recognized that many of the features of celiac disease may not be explained on the basis of malabsorption alone. It is proposed that the hypersensitivity to gluten is not limited to intestine alone; many other organs are affected independently of intestinal involvement and celiac disease is now considered as a systemic disease. Furthermore, celiac disease is not only one disease caused by gluten; in fact there is a wide spectrum of gluten related diseases. Gluten-related disorders have been classified recently as allergic (wheat allergy), autoimmune (celiac disease, dermatitis herpetiformis, and gluten ataxia), and possibly immune-mediated (gluten sensitivity).

In this special issue on celiac disease, five articles have highlighted various aspects of celiac disease. Approximately 30–50% of patients with celiac disease present to clinicians with features other than gastrointestinal manifestations such as with short stature, hypothyroidism, or type I diabetes to endocrinologists; with refractory anemia to hematologists; or with infertility or delayed menarche to gynecologists. Emami M. H. and coauthors from Iran in this issue have reported celiac disease in 5.9% (9/151) and 1.25% (3/173) of patients presenting with typical and atypical features of malabsorption. B. Admou et al. from Morocco have discussed in a review article about the atypical presentation of celiac disease and how to manage them.

Nine to 72% of patients with celiac disease have low bone mineral density (BMD). On the other hand 4.5–12% of patients having osteoporosis/osteopenia had a positive celiac serology. The low BMD does not always normalize even after years of GFD. This observation suggests that other than malabsorption of calcium and phosphate, other mechanisms are also involved. In an article on bone involvement in celiac disease, T. Larussa et al. from Catanzaro, Italy, have discussed epidemiology, mechanisms, and treatment of bone involvement in patients with celiac disease.

Dermatitis herpetiformis is a cutaneous manifestation of small intestinal immune mediated enteropathy precipitated by exposure to dietary gluten. It is characterised by herpetiform clusters of pruritic urticated papules and vesicles on the skin, especially on the elbows, buttocks and knees, and IgA deposits in the dermal papillae. While dermatitis herpatiformis is a characteristic gluten-induced skin disease; there are other dermatological manifestations of celiac disease such as vitiligo, alopecia areata, psoriasis, urticaria, atopic dermatitis, and cutaneous vasculitis. C. Marzia et al. from Florence, Italy, have discussed in this issue about the dermatological manifestations of celiac disease.

The management of celiac disease is very different from other gastrointestinal diseases and the core of the treatment is dietary and nonmedicinal. The most effective, most safe and most affordable treatment of celiac disease at present is GFD. While prescribing GFD is easy, both institution and maintenance of compliance to GFD are challenging. The key to success is counseling by a nutrition specialist and maintenance of compliance by the patient. All foods and drugs that contain gluten and its derivatives must be eliminated from the diet because even 50 mg of gluten is sufficient to cause a significant increase in the intestinal mucosal damage.

 Patients with celiac disease are more prone to physical, psychological, and social strains than healthy children, which influence the patients health-related quality of life (HRQOL) not only because of disease but also because of restrictions caused by GFD. It can be hard for children, adolescents, or the adult patients with celiac disease to accept and comply with the strict diet. Such an experience to patients with celiac disease affects their quality of life. I. M. Byström et al. from Sweden, in this issue, have described the HRQOL in children and adolescents with celiac disease from the perspectives of children and parents. Children diagnosed before the age of five had higher HRQOL than children diagnosed later. Children who had the classical symptoms of the disease at onset scored better on HRQOL scales than those who had atypical symptoms or were asymptomatic. Moreover, parents of children with celiac disease scored the HRQOL of their children lower compared to that assessed by children themselves.

Celiac disease is one of the ten diseases which are often missed by doctors. Although absolute number of patients with celiac disease at present is not very large, the absolute number is however expected to increase markedly all over the world during the next decade. There are many issues which require immediate attention. The foremost of them include increasing the awareness about the disease amongst doctors and general population. A due emphasis on celiac disease should be placed during undergraduate and postgraduate curriculum. Furthermore, a constant reminder about this disease should be provided to the physicians, internists, gastroenterologists, hematologists, and endocrinologists through continuing medical education.

*Govind K. Makharia* *Govind K. Makharia* *Carlo Catassi* *Carlo Catassi* *Kheen Lee Goh* *Kheen Lee Goh* *C. J. J. Mulder* *C. J. J. Mulder*

